# Hepatoprotective Effect of *Terminalia chebula* against *t*-BHP-Induced Acute Liver Injury in C57/BL6 Mice

**DOI:** 10.1155/2015/517350

**Published:** 2015-01-26

**Authors:** Min-Kyung Choi, Hyeong-Geug Kim, Jong-Min Han, Jin-Seok Lee, Jong Suk Lee, Sun Ho Chung, Chang-Gue Son

**Affiliations:** ^1^Liver and Immunology Research Center, Daejeon Oriental Hospital of Daejeon University, 22-5 Daehung-dong, Jung-gu, Daejeon 301-724, Republic of Korea; ^2^GyeongGi Bio-Center, GSTEP, 864-1 Iui-dong, Yeongtong-gu, Suwon, Gyeonggi-do 443-270, Republic of Korea

## Abstract

We aimed to identify the hepatoprotective effects of *Terminalia chebula* water extract (TCW) and its corresponding pharmacological actions using C57/BL6 mice model of *tert*-butylhydroperoxide-(*t*-BHP-) induced acute liver injury. Mice were orally administered with TCW (0, 50, 100, or 200 mg/kg) or gallic acid (100 mg/kg) for 5 days before *t*-BHP (2.5 mM/kg) injection. Liver enzymes, histopathology, oxidative stress parameters, antioxidant components, and inflammatory cytokines were examined 18 h after *t*-BHP injection. *t*-BHP injection caused dramatic elevation of serum AST, ALT, and LDH level, while TCW pretreatment notably attenuated these elevations. Inflammatory cytokines including TNF-*α*, IL-1*β*, and IL-6 were notably increased in hepatic tissues, and then these were efficiently attenuated by TCW pretreatment. *t*-BHP injection notably increased malondialdehyde, total reactive oxygen species, and nitric oxide in the liver tissue, while it markedly dropped the antioxidant activities including total antioxidant capacity, total glutathione contents, glutathione peroxidase, superoxide dismutase, and catalase. TCW pretreatment remarkably ameliorated these alterations, and these effects were relevant to gene expressions. Histopathological examinations supported the above findings. Collectively, these findings well prove that TCW beneficially prevents acute and severe liver injury and clarify its corresponding mechanisms involved in the inhibition of oxidative stress and inflammatory cytokines.

## 1. Introduction

Liver is the largest organ which can be damaged by numerous causes including pathogen infections, harmful chemicals, and alcohol or drug abuse [1]. Liver is well-known to have a high potential of regeneration and recovery from injury [2]. Rarely, severe and acute case of liver injury can lead to life-threatening clinical syndromes including jaundice, severe coagulopathy, and high rates of mortality. There are still medical tasks for elucidating its pathophysiological mechanisms and development of efficient therapy for especially severe hepatic injury [3].

Previous studies well reported that the oxidative stress is closely linked to the pathogenesis of acute liver injury [4]. The high level of reactive oxygen species (ROS) can directly damage liver tissue via impairment of cellular macromolecules and structures [5]. Moreover, the excessive ROS can lead to alteration of cellular membrane permeability, activation of proteases and nucleases, and deoxyribonucleic acid (DNA) fragmentations in hepatic tissue [6]. Therefore the oxidative stress and antioxidant activities are critical issues on development of the hepatoprotective or therapeutics drug recently [7–9].

Based on the traditional use and clinical experiences, many groups have tried to develop hepatotherapeutic agents using herbal plants [10, 11].* Terminalia chebula* (Chebulae Fructus) belongs to Combretaceae family, which is originated from India. The dried type of maturated* Terminalia chebula* fruit is used to treat various disorders including urinary tract diseases, dermatitis, diabetes mellitus, cardiovascular syndromes, and hepatic disorders [12–14]. Some studies partially showed the hepatoprotective and antioxidant effects of* Terminalia chebula* in chronic animal models [15, 16].

Most of them were conducted in chronic hepatic injury models, but only one study was done using a* t*-BHP-induced acute liver injury model [17]. According to this study, however, hepatoprotective effects were evaluated at a very high dose of* Terminalia chebula* (500 or 1000 mg/kg) under the mild hepatic injury condition which was induced by 0.1 mM/kg of* t*-BHP-treatment.

In the current study, therefore, we aimed to evaluate the hepatoprotective efficacy of* Terminalia chebula* under severe acute liver injury condition induced by high concentration of* t*-BHP-treatment (2.5 mM/kg) and relatively low dose of* Terminalia chebula* and to explore its corresponding mechanisms.

## 2. Material and Methods

### 2.1. Reagents and Chemicals

The chemicals, gallic acid,* tert*-butylhydroperoxide (*t*-BHP), potassium chloride (KCl), 1,1,3,3-tetraethoxypropane (TEP), ferrous sulfate, 4-amino-3-hydrazino-5-mercapto-1,2,4-triazole (Purpald), myoglobin, 2,2′-azino-bis(3-ethylbenzothiazoline-6-sulfonic acid) diammonium salt (ABTS), reduced glutathione (GSH), 5,5-dithiobis-(2-nitrobenzoic acid) (DTNB), glutathione reductase (GSH-reductase), and *β*-nicotinamide adenine dinucleotide phosphate-reduced form (*β*-NADPH) were all purchased from Sigma (MO, USA); and thiobarbituric acid (TBA) was obtained from Lancaster Co. (Lancashire, England). Hydrogen peroxide (H_2_O_2_) was purchased from Junsei Chemical Co., Ltd. (Tokyo, Japan). Dimethyl sulfoxide (DMSO), phosphoric acid (H_3_PO_4_), formic acid, acetonitrile, methanol, and *n*-butanol were purchased from J. T. Baker (Mexico City, Mexico), and 1 M Tris–HCl solution (pH 7.4) and 500 mM ethylenediaminetetraacetic acid (EDTA) solution (pH 8.0) were purchased from Bioneer (Daejeon, Republic of Korea). Mayer's hematoxylin was purchased from Sigma-Aldrich (St. Louis, MO).

### 2.2. Preparation of* Terminalia chebula* Water Extract (TCW) and Fingerprinting Analysis

*Terminalia chebula* was purchased from an herbal pharmaceutical company (Jeong-Seong Drugstore, Daejeon, Republic of Korea).* Terminalia chebula* was identified by a professor with a herbology specialty in the Oriental Medical College of Daejeon University. One hundred grams of* Terminalia chebula* was boiled in 1 L of distilled water (DW) for 90 min using automatic nonpressure pot (Dae-Woong, Seoul, Republic of Korea). The extract was centrifuged for 15 min at 150 ×g, and the supernatant was lyophilized using vacuum freeze drying system and stored at −20°C for using. The final extraction yield was 11.58% (w/w).

Fingerprinting for the reproducibility of the* Terminalia chebula* water extract (TCW) and compositional analysis of putative compounds were conducted using ultra-high-performance liquid chromatography-tandem mass spectrometry (UHPLC-MS/MS). A 5 mg aliquot of the TCW sample was dissolved in 1 mL of 90% methanol, and the solution was filtered (0.45 *μ*m). The 10 *μ*L of TCW sample solution was subjected to UHPLC-MS using an LTQ Orbitrap XL linear ion-trap MS system (Thermo Scientific Co., San Jose, CA, USA) equipped with an electrospray ionization source. Separation was performed on an Accela UHPLC system using an Acquity BEH C18 column (1.7 *μ*m, 100 × 2.1 mm; Waters, Milford, MA, USA). The column was eluted at a flow rate of 0.4 mL/min using water (in 0.1% formic acid) and acetonitrile (in 0.1% formic acid), which were used as mobile phases A and B, respectively, with the following gradients: 0-1 min, 0-1% B in A; 1–7 min, 1–100% B in A; 7–10 min, 100–1% B in A (linear gradient). The compositional analysis of TCW was conducted using a photodiode array at 200–600 nm. The full-scan mass spectra were acquired at 150–1500 *m*/*z* in positive and negative modes. An Orbitrap analyzer was used for high-resolution mass data acquisition with a mass resolving power of 30,000 FWHM at 400 *m*/*z*. Tandem mass (MS/MS) spectra were acquired in data-dependent mode by collision-induced dissociation.

For quantitative analysis of gallic acid, a well-known reference compound of* Terminalia chebula*, the calibration curve was calculated three times using UHPLC-MS with a Quattro Micro triple quadrupole mass spectrometer coupled with an Acquity ultra performance liquid chromatography (Waters, Ireland). The mass spectrometer was equipped with electrospray ionization in the negative mode. Separation conditions including the mobile phase conditions and other conditions were as described above. All aspects of system operation and data acquisition were controlled using Mass Lynx software (see Supplementary Figure 1 in Supplementary Material available online at http://dx.doi.org/10.1155/2015/517350).

### 2.3. Animals and Experimental Design

For the experiment, 36 heads of specific pathogen-free male C57BL/6N mice (6 weeks old, 18–20 g) were obtained from commercial animal breeder (Dae Han Bio Link, Chungbuk, Republic of Korea) and acclimated for l week before experiment. All animals were housed in an environmentally controlled room at 22 ± 2°C and 60 ± 5% relative humidity under 12/12 h light/dark cycle. All mice were freely fed commercial pellets (G-Bio, Gyeonggi, Republic of Korea) with tap water* ad libitum*. All of the mice were randomly divided into six groups (*n* = 6 for each group): naive (distilled water without* t*-BHP injection), control (distilled water and* t*-BHP injection), TCW treatment groups (50, 100, or 200 mg/kg of TCW and* t*-BHP injection), and positive control group (100 mg/kg of gallic acid and* t*-BHP injection), respectively. The TCW and gallic acid were dissolved in distilled water. The distilled water, TCW, or gallic acid was orally administrated for 5 consecutive days before* t*-BHP injection. Two hours after the final drug treatment, severe acute liver injury was induced by intraperitoneal injection with* t*-BHP (dissolved in normal saline as 2.5 mM/kg). The naive group was intraperitoneally injected normal saline. All mice were starved for 18 h after* t*-BHP or saline injection, and then they were sacrificed by collection of whole blood via an abdominal vein under ether anesthesia. Liver tissue was removed and immediately weighed and was fixed or stored in 10% neutral buffered formalin, RNAlater (Ambion, TX, USA), or at deep freezer (−70°C) for histopathological analysis, mRNA expression analysis, and determination of biochemical parameters, respectively.

Animal experiments were conducted in accordance with the Guide for the Care and Use of Laboratory Animals published by the United States National Institutes of Health. The protocol was approved by the Institutional Animal Care and Use Committee of Daejeon University (approval number DJUARB: 2012-017).

### 2.4. Preparation of Hepatic Tissue Homogenate and Determination of Protein Content

For analysis of protein based parameters, the isolated liver tissues were homogenized in radio immune precipitation assay (RIPA) buffer and centrifuged at 10,000 ×g for 15 min, and protein content was determined using a Bicinchoninic Acid (BCA) Protein assay kit (Sigma, St. Louis, MO, USA). The supernatant was transferred and stored at −70°C until required. For determination of malondialdehyde (MDA), liver tissues (200 mg) were homogenized in ice-cold KCl (1.15%).

### 2.5. Serum Biochemical Analysis

Blood was collected from the abdominal vein. After centrifuging at 3,000 ×g for 15 min, the serum was separated and stored in −70°C. The serum levels of aspartate transaminase (AST), alanine transaminase (ALT), and lactate dehydrogenase (LDH) were determined using an Auto Chemistry Analyzer (AU400; Olympus, Tokyo, Japan).

### 2.6. Histopathological and Immunohistochemical Examination

The hepatic tissue fixed in 10% neutral buffered formalin was embedded in paraffin and cut into 5 *μ*m thick sections for histomorphological and immunohistochemical examination. After drying, hepatic tissue section slides were stained with hematoxylin and eosin (H&E) according to standard procedures. For immunohistochemistry, sections were incubated with 4-hydroxynonenal (4-HNE) primary antibody (1 : 200; Abcam, Cambridge, UK) and biotinylated secondary antibody (Nichirei Biosciences, Tokyo, Japan), followed by the avidin-biotin-peroxidase complex. The immunoreactive signal was developed by their substrates, DAB (3,3′-diaminobenzidine) and AEC (3-amino-9-ethylcarbazole) (Abcam). The slides were counterstained with Mayer's hematoxylin (Sigma-Aldrich, St. Louis, MO) and examined under an optical microscope (Leica Microsystems, Wetzlar, Germany).

### 2.7. Determination of Lipid Peroxidation in Hepatic Tissue

Lipid peroxidation levels were evaluated by measuring MDA content using the thiobarbituric acid reactive substances (TBARS) method, as described previously [18]. Briefly, the hepatic tissue homogenate (in the 1.15% of KCl buffer) was mixed with 1% H_3_PO_4_ and 0.67% TBA solution. The mixture was heated for 45 min at 100°C; *n*-butanol was added and followed by vigorous vortexing and centrifuged at 3,000 ×g for 15 min. The absorbance of the supernatant was measured at 535 and 520 nm and compared to a standard value (freshly prepared TEP solution).

### 2.8. Determination of ROS and Nitric Oxide (NO) in the Hepatic Tissue

The ROS assay was performed by staining with previous method with slight modifications [19]. In brief, 3 *μ*L hepatic tissue homogenates or standard solutions were transferred to the 96-well microplate. After then, 180 *μ*L of reagent mixture (*N,N*-diethyl-*para*-phenylenediamine 6 mg/mL with 4.37 *μ*M of ferrous sulfate dissolved in 0.1 M sodium acetate buffer, pH 4.8) was added to each well at 37°C for 5 min. The absorbance of the plate was measured at 505 nm using a microplate reader (Molecular Device Corp., Sunnyvale, CA, USA). ROS levels from hepatic tissue were calculated from a calibration curve of H_2_O_2_ and expressed as H_2_O_2_ equivalent (1 unit = 1.0 mg H_2_O_2_/L).

NO levels in hepatic tissue were determined by Griess's reagent [20]. Briefly, 40 *μ*L of hepatic homogenate was mixed with 160 *μ*L of Griess reagent (1% sulfanilamide, 0.1% N-(1-naphthyl) ethylenediamine hydrochloride, 2.5% H_3_PO_4_) in a 96-well plate, and the absorbance was then read at 540 nm with a microplate reader (Molecular Devices).

### 2.9. Determination of Total Antioxidant Capacity (TAC) in Hepatic Tissue

The TAC was determined using a previously described method [21]. Briefly, 90 *μ*L of phosphate-buffered saline (10 mM, pH 7.2), 50 *μ*L of myoglobin solution (18 *μ*M), 20 *μ*L of 3 mM ABTS solution, and 20 *μ*L of standard (gallic acid) or diluted hepatic tissue homogenate were added to a 96-well microplate and mixed well at 26°C for 3 min. Then, 20 *μ*L of H_2_O_2_ (250 *μ*M) was added to each well and the plate was incubated for 5 min. The absorbance at 600 nm was measured using a spectrophotometer (Molecular Devices). TAC was expressed as the gallic acid equivalent antioxidant capacity (GEAC).

### 2.10. Determination of GSH Contents, GSH-Peroxidase (GSH-Px), and GSH-Reductase (GSH-Rd) in Hepatic Tissue

GSH contents were determined with slight modification of previous method [22]. Briefly, 50 *μ*L of GSH standard or diluted hepatic tissue homogenate (in 10 mM PBS, pH 7.2) was combined with 80 *μ*L of DTNB/NADPH mixture (10 *μ*L of 4 mM DTNB and 70 *μ*L of 0.3 mM NADPH) in a 96-well microplate. Finally, 20 *μ*L (0.06 U) of GSH-Rd solution was added to each well, and then 405 nm was measured against a reagent blank after 5 min and used to calculate *μ*M GSH/mg protein.

The activity of GSH-Px was calculated by the method of the previously described method [23]. Briefly, 50 *μ*L of NADPH reagent (5 mM NADPH, 42 mM GSH, 10 U/mL GSH-Rd in 1.25 mL of distilled water) was added to 890 *μ*L of GSH-Px buffer (50 mM Tris HCl, pH 8.0, containing 0.5 mM EDTA). Then, 50 *μ*L of diluted hepatic tissue homogenate and 10 *μ*L of 30 mM* t*-BHP solution were added to the mixture. The absorbance at 340 nm was finally measured using a UV-visible spectrophotometer (Varian, Agilent Technologies, Santa Clara, CA, USA). Enzyme activity was calculated using the following formula: enzyme activity (U/mL) = (Δ*A*_340_ × dilution factor)/(6.22 × sample volume in mL).

GSH-Rd activity in hepatic tissue was determined using a previously described method [24, 25]. Briefly, 150 *μ*L of GSSG and 30 *μ*L of GSH-Rd assay buffer (100 mM potassium phosphate buffer, pH 7.5, containing 1 mM EDTA) were added to 30 *μ*L of hepatic tissue homogenate and diluted with GSH-Rd dilution buffer (100 mM potassium phosphate buffer, pH 7.5, containing 1 mM EDTA, and 1 mg/mL bovine serum albumin). Then, at the addition of 75 *μ*L DTNB and 2 mM NADPH, the absorbance at 412 nm was measured using a spectrophotometer (Molecular Devices). Enzyme activity was calculated using the following formula: enzyme activity (U/mL) = {(Δ*A*_sample_ − Δ*A*_blank_)×(dilution factor)}/{*ε*mM × (volume of sample in mL)}, where *ε*mM is equal to 14.15 mM^−1^ cm^−1^.

### 2.11. Determination of Superoxide Dismutase (SOD) and Catalase (CAT) Activities in Hepatic Tissue

SOD activity in hepatic tissue was determined using a SOD assay kit (Dojindo Laboratories, Kumamoto, Japan), according to the manufacturer's protocol. Bovine erythrocyte SOD (Sigma, MO, USA) was used as a standard. 20 *μ*L of standard or diluted hepatic tissue homogenate was mixed with 200 *μ*L of WST-1 working solution in a 96-well plate. Subsequently, 20 *μ*L of enzyme working solution was added to each well and thoroughly mixed. The plate was incubated at 37°C for 20 min, and 450 nm was measured using a microplate reader (Molecular Devices).

CAT activity in the hepatic tissue was determined using the method of the previously described method [26]. Briefly, 140 *μ*L of phosphate buffer (250 mM, pH 7.0), 150 *μ*L of 12 mM methanol, and 30 *μ*L of H_2_O_2_ were mixed with 300 *μ*L of standard or diluted hepatic tissue homogenate in a 13 × 100 mm test tube. The reaction was allowed to proceed for 10 to 20 min and was stopped by the addition of 450 *μ*L of Purpald (22.8 mM in 2 N potassium hydroxide). The mixture was maintained for 20 min at 25°C, and then 150 *μ*L of potassium periodate (65.2 mM in 0.5 N potassium hydrate) was added to the same tube. The absorbance was measured at 550 nm using a microplate reader (Molecular Devices).

### 2.12. Determination of Tumor Necrosis Factor-Alpha (TNF-*α*), Interleukin-1*β* (IL-1*β*), and Interleukin-6 (IL-6) Levels in Hepatic Tissue

TNF-*α*, IL-1*β*, and IL-6 levels in hepatic tissue were measured using commercial ELISA kits according to the manufacturers' instructions (Biosource, Camarillo, CA, USA; R&D Systems, Minneapolis, MN, USA). The absorbance at 450 and 570 nm was measured using a spectrophotometer (Molecular Devices).

### 2.13. Gene Expression Analysis by Quantitative Real-Time PCR

Total RNA was extracted from hepatic tissue samples with Trizol reagent (Molecular Research Center, Cincinnati, OH). The cDNA was synthesized from total RNA (2 *μ*g) in a 20 *μ*L reaction using a High-Capacity cDNA Reverse Transcription Kit (Ambion, Austin, TX). The primers for GSH synthase (GSH-Sy), GSH-Px, GSH-Rd, SOD-1, SOD-2, SOD-3, CAT, inducible nitric oxide synthase (iNOS), TNF-*α*, IL-1*β*, IL-6, cytochrome P450 2E1 (CYP2E1), and *β*-actin were as follows (5′ → 3′, forward and reverse): GSH-Sy, 5′-TGT GCC CTT TTA CCC TCT TCC T-3′ and 5′-TCT TTG GAG TGT GGG AAT GGA-3′; GSH-Px, 5′-CTC ACC ATT CAC TTC GCA CTT C-3′ and 5′-ACA CCA GGA GAA TGG CAA GAA-3′; GSH-Rd, 5′-GGA AGC GTG ATG GAC TTG TAT AAA A-3′ and 5′-TGG AAT CTG CCT GAG AAG CA-3′; SOD-1, 5′-CAG CCT TGT GTA TTG TCC CCA TA-3′ and 5′-CAG AAG GCA AGC GGT GAA C-3′; SOD-2, 5′-GGT GGC GTT GAG ATT GTT CA-3′ and 5′-CCC AGA CCT GCC TTA CGA CTA T-3′; SOD-3, 5′-AAA GGT TCC CAA ATA CTC TCT CTA AGG-3′ and 5′-CCC ACC CCC AAG TTC CAT-3′; CAT, 5′-GAA TCC GCT CTC TGT CAA AGT GT-3′ and 5′-GGA GGC GGG AAC CCA ATA-3′; iNOS, 5′-CTT TAG ACC TCA ACA GAG CC-3′ and 5′-GTA GGA CAA TCC ACA ACT CG-3′; TNF-*α*, 5′-CTC AGA TCA TCT CAA AAT TCG AGT A-3′ and 5′-CTT CAC AGA GCA ATG ACT CCA AAG T-3′; IL-1*β*, 5′-CAT TGA GGT GGA GAG CTT TC-3′ and 5′-ATG AGG ACA TGA GCA CCT TC-3′; IL-6, 5′-GCT ACC TGG AGT ACA TGA AG-3′ and 5′-CTG TGA CTC CAG CTT ATC TG-3′; CYP2E1, 5′-AGA TAA TCC GCA AAG TTA TTG TAA AGC-3′ and 5′-TGA CAA GAA GTG TCT GAG GCT CAT-3′; and *β*-actin, 5′-GC ACC ACA CCT TCT ACA ATG A-3′ and 5′-ATC TTT TCA CGG TTG GCC TTA G-3′. Real-time PCR reactions were conducted using PikoReal Real-Time PCR System (Thermo Scientific, CA, USA) and performed according to the manufacturer's protocol. Reactions were performed in a total volume of 10 *μ*L, containing 1.5 *μ*L of cDNA, 5 *μ*L of SYBR Green qPCR Master Mix (K0259, Thermo Scientific, CA, USA), 0.5 *μ*L of each primer, and 3 *μ*L of water (R0581, Thermo Scientific, CA, USA). PCR amplification cycles were carried out as follows: 30 s at 98°C, 40 cycles of 5 s at 98°C, 10 s at 65°C, and 30 s at 72°C. For each sample, two reactions were performed at the same time. One reaction was performed to determine the mRNA level of the target gene, and the second was performed to determine level of *β*-actin.

### 2.14. Statistical Analysis

The results were expressed as the mean ± standard deviation (*n* = 6). Differences between groups were analyzed using a one-way analysis of variance (ANOVA) followed by the least significant difference post hoc test. In all analyses, the values of *P* < 0.05 were considered statistically significant.

## 3. Results

### 3.1. Chemical Compositions of TCW

**Figure 1:**
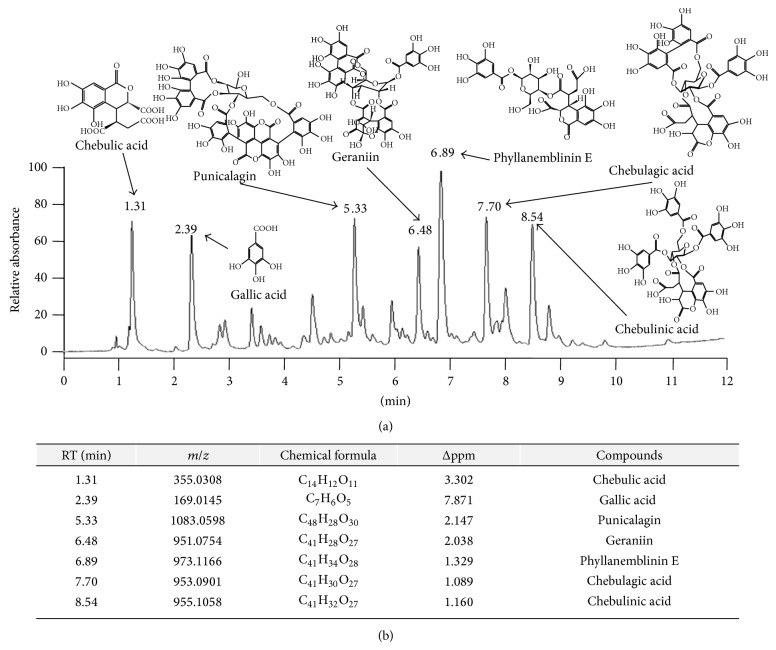
Ultra-high-performance liquid chromatography-tandem mass spectrometry (UHPLC-MS/MS) analysis of* Terminalia chebula* water extract (TCW). The TCW sample was subjected to UHPLC analysis (a), and seven main compounds were identified using an HPLC-MS database (b).

The chemical compounds from TCW were analyzed using UHPLC-MS/MS. A total of seven of the major peaks appeared at 1.31, 2.39, 5.33, 6.48, 6.89, 7.70, and 8.54 min of retention time, respectively (Figure 1(a)). From the chemical formulae analysis using high-resolution mass spectra, these compounds were identified as the chebulic acid (C_14_H_12_O_11_), gallic acid (C_7_H_6_O_5_), punicalagin (C_48_H_28_O_30_), geraniin (C_41_H_28_O_27_), phyllanemblinin E (C_41_H_34_O_28_), chebulagic acid (C_41_H_30_O_27_), and chebulinic acid (C_41_H_32_O_27_) (retention times at 1.31, 2.39, 5.33, 6.48, 6.89, 7.70, and 8.54, resp., Figure 1(b)). In addition, the quantitative analysis result showed that gallic acid content in TCW sample was approximately 1.69% (Supplementary Figure 1(c)).

### 3.2. Effect on the Serum Biochemistries

**Figure 2:**
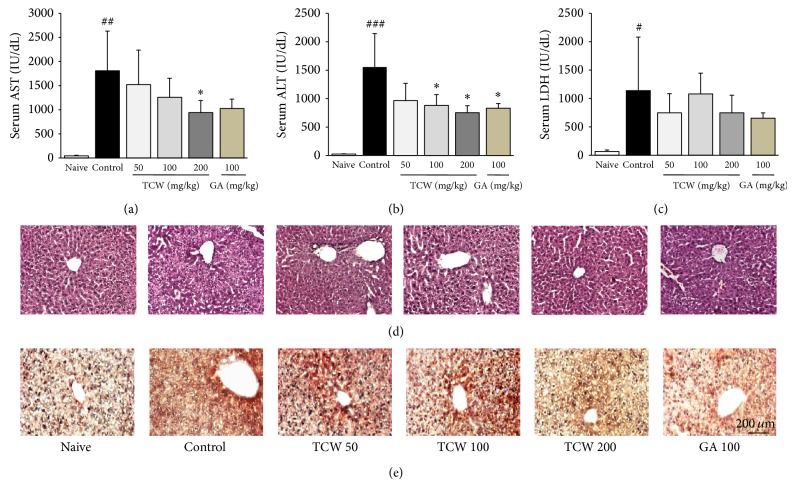
Serum biochemistries and histopathological and immunohistochemical findings of hepatic tissue. The mice were administered with TCW (50, 100, and 200 mg/kg), gallic acid, or distilled water orally once per day for 5 days. After 18 h of* t*-BHP injection, serum AST (a), ALT (b), and LDH (c) levels were measured. H&E staining (d) and anti-4-HNE staining (e) were conducted and examined under microscopy (×200). Data are expressed as the mean ± SD (*n* = 6). ^#^ *P* < 0.05, ^##^ *P* < 0.01, and ^###^ *P* < 0.001 compared with the naive group; ^*^ *P* < 0.05 compared with the control group.

*t*-BHP injection dramatically increased serum AST, ALT, and LDH levels approximately 41.7-, 62.2-, and 17.1-fold compared with naive group, whereas TCW pretreatment significantly attenuated these increases of both serum AST and ALT levels as compared with the control group, respectively (*P* < 0.05 for 200 mg/kg in AST; *P* < 0.05 for 100 and 200 mg/kg in ALT, Figures 2(a) and 2(b)). TCW pretreatment reduced serum LDH level but did not reach the statistical significance (Figure 2(c)). Gallic acid pretreatment showed similar effects as those of TCW.

### 3.3. Effects on the Histological Findings and Immunohistochemical Analysis

*t*-BHP injection caused remarkable hepatocyte necrosis and inflammatory cells infiltrations in the hepatic tissue as compared with naive group. Pretreatment with TCW, however, efficiently prevented both hepatocyte destructions and inflamed cell infiltrations in the hepatic tissue as shown by H&E staining compared with control group, respectively (Figure 2(d)). Immunohistochemistry result showed that the 4-HNE signals (part of dark brown color), as a potent marker of lipid peroxidation, were strongly enhanced after* t*-BHP injections as compared with the naive group, whereas those alterations were remarkably ameliorated by pretreatment with TCW (Figure 2(e)). Gallic acid also improved both histopathological and immunohistochemistry findings.

### 3.4. Effect on the MDA, ROS, and NO in Hepatic Tissue Levels

*t*-BHP injection considerably increased MDA level in the hepatic tissue by approximately 1.9-fold in naive group, whereas TCW pretreatment significantly attenuated the elevated MDA level compared with the control group, respectively (*P* < 0.05 for 100 and 200 mg/kg, Table 1).

The hepatic tissue levels of total ROS and NO were considerably increased by* t*-BHP injection up to 2.1- and 1.6-fold compared with the naive group, whereas TCW pretreatment significantly ameliorated these alterations compared with the control group (*P* < 0.05 for 200 mg/kg in NO, *P* < 0.01 for 200 mg/kg in ROS, resp., Table 1). Gallic acid pretreatment showed positive effect on only ROS level with statistical significance.

### 3.5. Effect on TAC and GSH Contents in Hepatic Tissue Levels

*t*-BHP injection significantly depleted hepatic tissue levels of both TAC and GSH contents (about 0.7-fold in both parameters) as compared with naive group. TCW pretreatment significantly reduced those deteriorations compared with the control group (*P* < 0.05 for 100 mg/kg in TAC, *P* < 0.01 for 200 mg/kg in TAC; for 50, 100, and 200 mg/kg in GSH, resp., Table 1). Gallic acid also had positive effects on both TAC and total GSH contents.

### 3.6. Effect on GSH-Px, GSH-Rd, SOD, and CAT Activities in Hepatic Tissues

*t*-BHP injection considerably decreased GSH-Px (but not GSH-Rd), SOD, and CAT activities in hepatic tissues compared with the naive group by approximately 0.7-, 0.8-, and 0.7-fold, respectively, whereas TCW pretreatment significantly attenuated these alterations, GSH-Px (*P* < 0.05 for 200 mg/kg), SOD (*P* < 0.05 for 50 mg/kg, *P* < 0.001 for 100 and 200 mg/kg), and CAT (*P* < 0.05, *P* < 0.01 for 100 mg/kg resp., Table 1). 200 mg/kg of TCW pretreatment significantly increased the hepatic tissue level of GSH-Rd (*P* < 0.05), and gallic acid pretreatment showed similar effects to TCW.

### 3.7. Effect on Proinflammatory Cytokines in Hepatic Tissues

**Figure 3:**
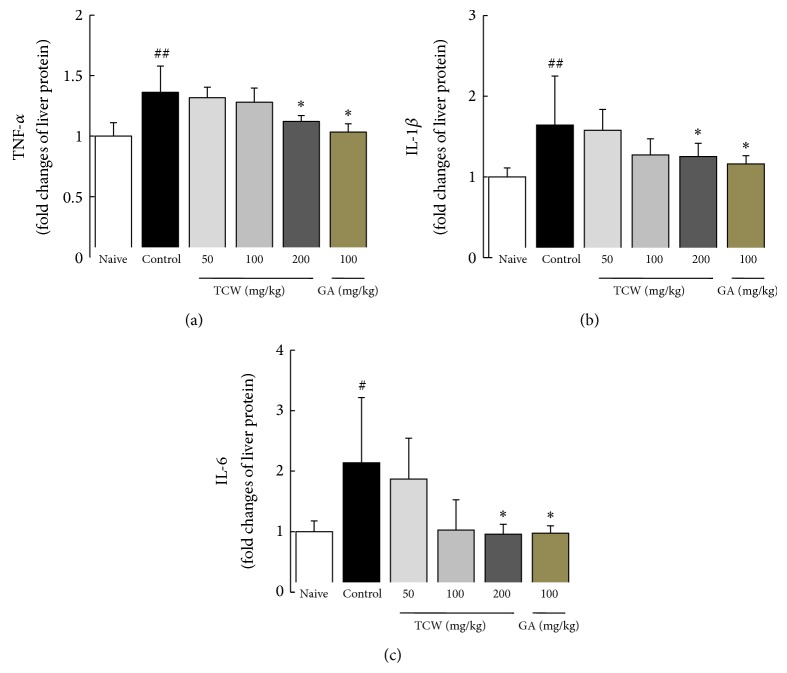
Cytokine changes in hepatic tissue. The mice were administered with TCW (50, 100, and 200 mg/kg), gallic acid, or distilled water orally once per day for 5 days. After 18 h of* t*-BHP injection, livers were removed and hepatic tissue levels of TNF-*α* (a), IL-1*β* (b), and IL-6 (c) were determined using a commercial ELISA kit. Data are expressed as the mean ± SD (*n* = 6). ^#^ *P* < 0.05 and ^##^ *P* < 0.01 compared with the naive group; ^*^ *P* < 0.05 compared with the control group.

*t*-BHP injection markedly elevated the hepatic tissue levels of proinflammatory cytokines, TNF-*α*, IL-1*β*, and IL-6 in hepatic tissue by 1.5, 1.7-, and 2.1-fold as compared with the naive group, respectively. TCW pretreatment (especially 200 mg/kg) significantly ameliorated these elevations in three cytokines as compared with control group (*P* < 0.05 in TNF-*α*, IL-1*β*, and IL-6, Figures 3(a) to 3(c)). Gallic acid pretreatment caused moderate reductions in TNF-*α* and IL-1*β*, but not IL-6 level.

### 3.8. Effects on Gene Expression in Hepatic Tissues

**Figure 4:**
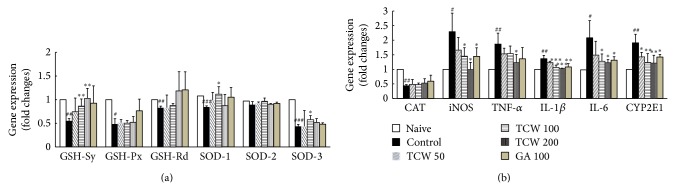
Antioxidant enzyme and proinflammatory and anti-inflammatory-related genes expression in hepatic tissue. The mice were administered with TCW (50, 100, and 200 mg/kg), gallic acid, or distilled water orally once per day for 5 days. After 18 h of* t*-BHP injection, livers were removed and mRNA levels of GSH-Sy, GSH-Px, GSH-Rd, SOD-1, SOD-2, and SOD-3 (a) and CAT, iNOS, TNF-*α*, IL-1*β*, IL-6, and CYP2E1 (b) in hepatic tissue were determined using real-time PCR. The mRNA levels were normalized to that of *β*-actin. Data are expressed as the mean ± SD (*n* = 6). ^#^ *P* < 0.05, ^##^ *P* < 0.01, and ^###^ *P* < 0.001 compared with the naive group; ^*^ *P* < 0.05, ^**^ *P* < 0.01, and ^***^ *P* < 0.001 compared with the control group.

*t*-BHP injection remarkably downregulated the antioxidant-related gene expressions including GSH-Sy (0.5-fold), GSH-Px (0.5-fold), GSH-Rd (0.8-fold), SOD-1 (0.8-fold), SOD-2 (0.9-fold), SOD-3 (0.4-fold), and CAT (0.4-fold) compared with the naive group, respectively. TCW pretreatment significantly normalized the gene expression of especially GSH Sy (*P* < 0.01 for 100 and 200 mg/kg). Other gene expressions (except SOD-1 and SOD-3 *P* < 0.05 for 100 mg/kg) were not significantly modulated (Figure 4(a)).

The inflammatory-related gene expressions including iNOS (2.3-fold), TNF-*α* (1.9-fold), IL-1*β* (1.4-fold), and IL-6 (2.1-fold) were markedly upregulated compared with the naive group, respectively. These alterations were significantly normalized by TCW pretreatment (*P* < 0.05 for 100 and 200 mg/kg in iNOS; *P* < 0.05 for 200 mg/kg in TNF-*α*; *P* < 0.001 for 100 and 200 mg/kg in IL-1*β*; *P* < 0.05 for 100 and 200 mg/kg in IL-6, resp., Figure 4(b)). TCW pretreatment significantly normalized the abnormal upregulation of CYP2E1 in gene expressions level (*P* < 0.05 for 50 mg/kg, *P* < 0.01 for 100 and 200 mg/kg, resp., Figure 4(b)). Gallic acid pretreatment did not affect antioxidant-related gene expressions, whereas it considerably normalized the abnormal gene expressions of iNOS, IL-1*β*, IL-6, and CYP2E1, respectively.

## 4. Discussion

Whatever the reason of liver injury is, certain degrees of damage in liver tissue could be recovered. In the case of the acute and severe liver injury, however, this status is sometimes difficult to be controlled [27]. In order to investigate the hepatoprotective activity of TCW in severe and acute hepatic injury condition, we adapted a toxic injury model using* t*-BHP. As per our expectation,* t*-BHP injection extremely damaged the liver tissue with the dramatic elevations of serum biochemical parameters including AST, ALT, and LDH (Figures 2(a) and 2(b)). The histopathological features of hepatic tissue also showed the signs of rigorous necrosis or inflammatory cell infiltration in hepatic tissues (Figure 2(d)).

Among many pathophysiological mechanisms of hepatic damage, oxidative stress is well-known to play a key role in the process of acute liver injuries [28]. This* t*-BHP-induced acute and severe liver injury model has been commonly used in recent days, which initiates oxidative stress damage to cellular levels [29]. During the metabolic process of* t*-BHP into* tert-*butanol, free radical intermediates are readily generated via activation of phase I enzymes, especially cytochrome P450 in liver [30, 31]. These intermediates deplete antioxidant enzymes including glutathione redox and sequentially generate oxidative stress leading to hepatic damage [6]. Our results showed that* t*-BHP injection increased the hepatic tissue levels of ROS, NO, and MDA content, a final product of lipid peroxidation (Table 1). These oxidative stress statuses were also confirmed by immunohistochemistry using the 4-HNE, known as a marker of protein oxidation product (Figure 2(e)). TCW pretreatment efficiently protected liver tissue from oxidative injury which was evidenced by normalizations of liver enzymes, oxidative stress parameters, and histological examination of serum or hepatic tissue (Figures 2(a) to 2(e) and Table 1).

Oxidative stress is referred to as the imbalance between oxidative stressors and antioxidant activities. To defend oxidative stress-mediated tissue damage, the antioxidant system has been well equipped in all of the biological organisms [11, 32]. Thus many of hepatotherapeutic candidates have focused on the activation of antioxidant capacities [33, 34]. As a regulator of intracellular redox homeostasis, GSH is well-known as a potent antioxidant protein and it is ubiquitously presented in every cell type [35]. Additionally, enzymatic-antioxidants including SOD, CAT, GSH-Rd, and GSH-Px exert to defend the oxidative damage via sequestrating the oxidative stressors [36]. In this study,* t*-BHP injection depleted antioxidant components including GSH contents and TAC levels and antioxidant enzymes' (SOD, CAT, GSH-Rd and GSH-Px) activities in both protein and gene expression levels of hepatic tissue. We observed, however, that the TCW pretreatment significantly blocked these deteriorations (Table 1 and Figure 4(a)).

Oxidative stress stimulates the inflammatory reaction via activation of proinflammatory cytokines including TNF-*α*, IL-1*β*, and IL-6 [27]. These proinflammatory cytokines play critical roles in the initiation and development of severe and acute liver injury [37, 38]. As per our expectation, the* t*-BHP injection considerably altered proinflammatory cytokines including TNF-*α*, IL-1*β*, and levels in both gene expression and protein levels in liver tissue. Additionally, the upregulated gene expression of iNOS in hepatic tissue was also significantly normalized by TCW pretreatment (Figures 3(a) to 3(c) and 4(b)). The modulation of iNOS is a main strategy in controlling the oxidative stress and inflammation-related diseases because it plays a central role in NO synthesis [39]. Our result also showed that TCW significantly normalized the abnormal level of CYP2E1 gene expression (Figure 4(b)). CYP2E1 is mainly activated and formed by the protein adducts during drug metabolism in liver, where protein adducts act as free radicals which highly react with the tissues, leading to acceleration of the tissue damage [40].

Previous studies well reported the gallic acid, gallate esters, and chebulic ellagitannins as the representative compounds of* Terminalia chebula* [41, 42]. Among them, gallic acid is most well-known for the potential antioxidant capacities as well as anti-inflammatory properties [43]. In this study, the content of gallic acid in TCW was only 1.69% (Supplementary Figure 1(c)). The hepatoprotective activity levels of 100 mg/Kg of gallic acid (as positive control) were equivalent to 200 mg/Kg of TCW. The compositional analysis presented the additional compounds including chebulic acid, punicalagin, geraniin, phyllanemblinin E, chebulagic acid, and chebulinic acid in TCW. Accordingly, we supposed that the corresponding activity of TCW might be owing to other compounds besides gallic acid. Among these compounds, chebulic acid had been studied for its antioxidant and hepatoprotective effects [44]. The chebulagic acid showed immune-modulation effects on IL-6 and IL-10 in collagen-induced arthritis model [45].

Three groups already have showed the hepatoprotective effects of* Terminalia chebula* by using animal models. 70% methanol and 95% ethanol extract of* Terminalia chebula* showed hepatoprotective effects in iron-dextran injection model [15] and antituberculosis drug-induced toxicity model [16], respectively. One group revealed the hepatoprotective effects of* Terminalia chebula*; however, the model involved as slight injury condition generated the very low dose of* t*-BHP (0.1 mM/kg) and provided the treatment with very high dose of* Terminalia chebula* (500 and 1000 mg/kg) [17]. In order to confirm the applicability of the potent hepatoprotective effects of* Terminalia chebula* against severe liver injury, our current study adapted the high dose of* t*-BHP (2.5 mM/kg) and provided the treatment with less doses of* Terminalia chebula* (50, 100, and 200 mg/kg), further investigating the possible pharmacological actions of* Terminalia chebula*.

## 5. Conclusion

Our results indicate that TCW efficiently protects the liver against acute and severe liver injury, and the underlying possible mechanisms may involve the enhancement of antioxidant capacities and modulation of inflammatory reactions.

## Supplementary Material

Supplementary Material: The Quantitative analysis of gallic acid content from TCW is presented.

## Figures and Tables

**Table 1:** Changes of oxidative stress and antioxidant system components.

Groups	Naive (fold)	Control (fold)	TCW (fold)			Gallic acid 100 mg/kg (fold)
			50 mg/kg	100 mg/kg	200 mg/kg	
MDA contents	11.7 ± 2.4	22.0 ± 8.1^#^	14.3 ± 3.3	12.3 ± 3.4^*^	10.2 ± 0.9^*^	13.8 ± 2.9
(*μ*M/mg protein)	(1.0 ± 0.2)	(1.9 ± 0.4)	(1.2 ± 0.2)	(1.0 ± 0.3)	(0.9 ± 0.1)	(1.2 ± 0.2)

Total ROS	21.2 ± 1.9	44.3 ± 9.1^##^	32.0 ± 9.3	35.1 ± 6.4	23.4 ± 5.5^**^	21.5 ± 4.2^**^
(U/mg protein)	(1.0 ± 0.1)	(2.1 ± 0.2)	(1.4 ± 0.3)	(1.7 ± 0.2)	(1.1 ± 0.2)	(1.0 ± 0.2)

NO	10.1 ± 0.6	16.1 ± 6.3^#^	13.5 ± 2.3	11.3 ± 0.8	10.6 ± 0.8^*^	12.9 ± 2.3
(*μ*M/mg protein)	(1.0 ± 0.1)	(1.6 ± 0.4)	(1.3 ± 0.2)	(1.1 ± 0.1)	(1.0 ± 0.1)	(1.3 ± 0.2)

TAC	636.3 ± 158.4	443.7 ± 87.7^#^	557.3 ± 127.2	721.4 ± 126.1^*^	776.4 ± 200.8^**^	742.2 ± 139.8^**^
(*μ*M/mg protein)	(1.0 ± 0.2)	(0.7 ± 0.2)	(0.9 ± 0.2)	(1.1 ± 0.2)	(1.2 ± 0.3)	(1.2 ± 0.2)

Total GSH	85.8 ± 6.2	59.9 ± 18.3^##^	86.2 ± 7.2^**^	83.9 ± 7.9^**^	85.9 ± 4.3^**^	81.7 ± 6.8^*^
(uM/mg protein)	(1.0 ± 0.1)	(0.7 ± 0.3)	(1.0 ± 0.1)	(1.0 ± 0.1)	(1.0 ± 0.1)	(1.0 ± 0.1)

GSH-Px	53.9 ± 11.4	36.0 ± 5.3^##^	45.5 ± 10.2	40.6 ± 8.3	47.2 ± 7.7^*^	42.9 ± 21.1
(U/mg protein)	(1.0 ± 0.2)	(0.7 ± 0.3)	(0.8 ± 0.3)	(0.8 ± 0.2)	(0.9 ± 0.2)	(0.8 ± 0.5)

GSH-Rd	37.4 ± 3.6	32.4 ± 6.2	46.8 ± 15.6	38.6 ± 17.1	51.1 ± 17.1^*^	45.0 ± 7.7^*^
(U/mg protein)	(1.0 ± 0.1)	(0.9 ± 0.2)	(1.3 ± 0.3)	(1.0 ± 0.4)	(1.4 ± 0.3)	(1.2 ± 0.2)

SOD activity	22.3 ± 3.5	18.3 ± 1.4^#^	29.2 ± 8.3^*^	27.4 ± 3.1^***^	29.8 ± 2.5^***^	25.9 ± 3.4^**^
(U/mg protein)	(1.0 ± 0.2)	(0.8 ± 0.1)	(1.3 ± 0.3)	(1.2 ± 0.1)	(1.3 ± 0.1)	(1.2 ± 0.1)

CAT activity	927.1 ± 145.7	681.4 ± 141.8^#^	972.9 ± 324.4	1111.2 ± 183.9^**^	985.1 ± 201.0^*^	895.7 ± 318.8
(U/mg protein)	(1.0 ± 0.2)	(0.7 ± 0.2)	(0.7 ± 0.3)	(1.2 ± 0.2)	(1.1 ± 0.2)	(1.0 ± 0.4)

Data were expressed as the mean ± SD (*n* = 6). ^#^ *P* < 0.05 and ^##^ *P* < 0.01 compared with the naive group; ^*^ *P* < 0.05, ^**^ *P* < 0.01, and ^***^ *P* < 0.001 compared with the control group. The figures shown in parentheses were normalized by each value of the naive group.

## Abbreviations

ALT: Alanine transaminase
AST: Aspartate transaminase
CAT: Catalase
CYP2E1: Cytochrome P450 2E1
GSH: Glutathione
GSH-Px: Glutathione peroxidase
GSH-Rd: Glutathione reductase
GSH-Sy: GSH synthase
H&E: Hematoxylin and eosin
IL-1*β*: Interleukin-1*β*
IL-6: Interleukin-6
iNOS: Inducible nitric oxide synthase
LDH: Lactate dehydrogenase
MDA: Malondialdehyde
NO: Nitric oxide
ROS: Reactive oxygen species
SOD: Superoxide dismutase
TAC: Total antioxidant capacity
*t*-BHP: * tert*-Butylhydroperoxide
TCW: * Terminalia chebula* water extract
TNF-*α*: Tumor necrosis factor-alpha
UHPLC: Ultra-high-performance liquid chromatography
4-HNE: 4-Hydroxynonenal.
