# Supplementary material for: Hepatoprotective Effect of Terminalia chebula against t-BHP-Induced Acute Liver Injury in C57/BL6 Mice
Source: Evid Based Complement Alternat Med. 2015 Jan 26;2015:517350. doi: 10.1155/2015/517350 (PMC4321673; doi:10.1155/2015/517350)

**Supplementary figure legend**

**Supplementary figure 1. Quantitative analysis of gallic acid content in TCW using UHPLC (Ultra-high-performance liquid chromatography)-MS.** TCW samples were dissolved in distilled water as 5 mg/mL and diluted it four doses (0.02 to 0.2 mg/mL) and get the linearity (A) and the peak areas of the TCW samples (B). The gallic acid content in TCW samples (C).

Supplementary Figure 1.


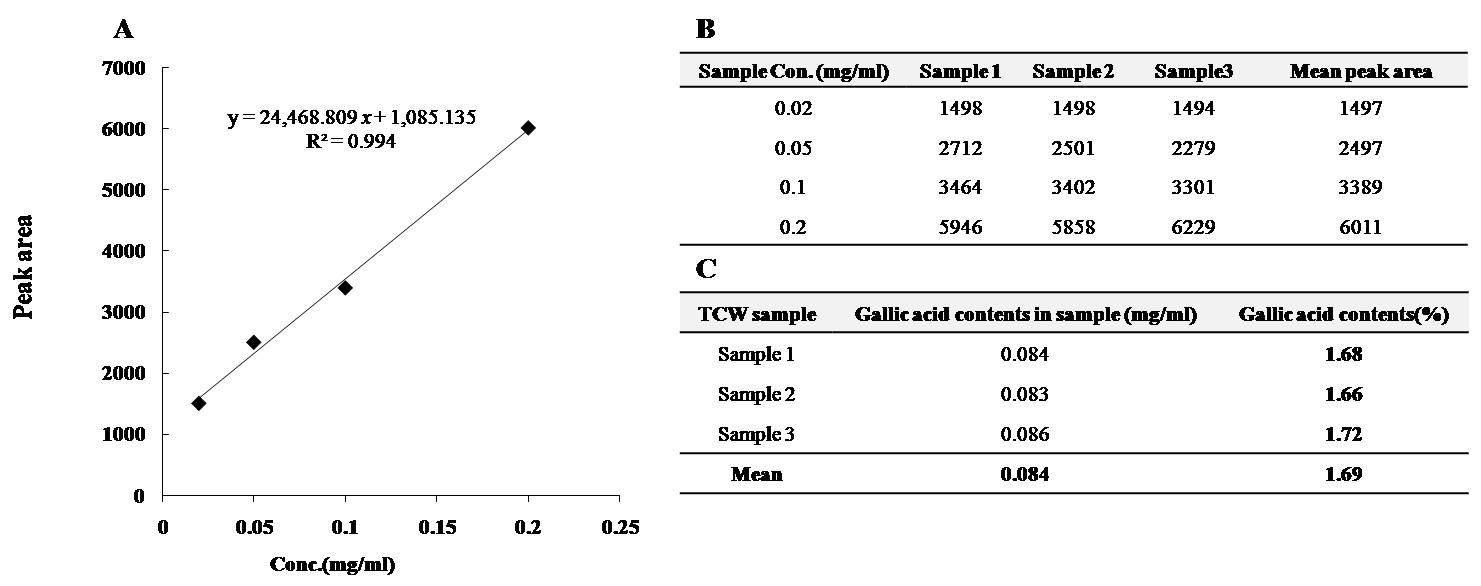

Supplement: Supplementary file 1 — Supplementary Material: The Quantitative analysis of gallic acid content from TCW is presented. [file 517350.f1.docx]
